# Microarray Analyses Demonstrate the Involvement of Type I Interferons in Psoriasiform Pathology Development in D6-deficient Mice*[Fn FN2]

**DOI:** 10.1074/jbc.M113.491563

**Published:** 2013-11-05

**Authors:** Helen M. Baldwin, Kenneth Pallas, Vicky King, Thomas Jamieson, Clive S. McKimmie, Robert J. B. Nibbs, José M. Carballido, Marcus Jaritz, Antal Rot, Gerard J. Graham

**Affiliations:** From the ‡ Chemokine Research Group, Institute of Infection, Immunity and Inflammation, University of Glasgow, Glasgow G12 8TA, Scotland, United Kingdom,; the § Beatson Institute for Cancer Research, Bearsden, Glasgow G61 1BD, United Kingdom,; the ¶ Novartis Institutes for Biomedical Research, Brunner Str. 59, A-1235 Vienna, Austria,; the ‖ Novartis Institutes for Biomedical Research, 4056 Basel, Switzerland, and; the ** University of Birmingham, Edgbaston, Birmingham B15 2TT, United Kingdom

**Keywords:** Chemokines, Cytokines/Interferon, Inflammation, Mouse, Psoriasis

## Abstract

The inflammatory response is normally limited by mechanisms regulating its resolution. In the absence of resolution, inflammatory pathologies can emerge, resulting in substantial morbidity and mortality. We have been studying the D6 chemokine scavenging receptor, which played an indispensable role in the resolution phase of inflammatory responses and does so by facilitating removal of inflammatory CC chemokines. In D6-deficient mice, otherwise innocuous cutaneous inflammatory stimuli induce a grossly exaggerated inflammatory response that bears many similarities to human psoriasis. In the present study, we have used transcriptomic approaches to define the molecular make up of this response. The data presented highlight potential roles for a number of cytokines in initiating and maintaining the psoriasis-like pathology. Most compellingly, we provide data indicating a key role for the type I interferon pathway in the emergence of this pathology. Neutralizing antibodies to type I interferons are able to ameliorate the psoriasis-like pathology, confirming a role in its development. Comparison of transcriptional data generated from this mouse model with equivalent data obtained from human psoriasis further demonstrates the strong similarities between the experimental and clinical systems. As such, the transcriptional data obtained in this preclinical model provide insights into the cytokine network active in exaggerated inflammatory responses and offer an excellent tool to evaluate the efficacy of compounds designed to therapeutically interfere with inflammatory processes.

## Teaser

**Background:** D6 regulates resolution of inflammatory responses. Its mode of action has not been molecularly defined.

**Results:** Microarray analysis of inflamed D6-deficient mouse skin identifies dysregulated type I interferon responses as underpinning exaggerated inflammatory responses in D6-deficient mice.

**Conclusion:** D6 is important for regulating type I interferon-based responses in inflammation.

**Significance:** The study provides novel insights into roles for D6 in the resolution of inflammatory responses.

## Introduction

Inflammatory responses are characterized by leukocyte migration to the inflamed site, a process ultimately dependent on chemokines and their receptors (1, 2). Chemokines are defined on the basis of the presence of a characteristic cysteine motif in their mature sequences, which is used to divide the chemokine family into four subfamilies. The two largest subdivisions comprise the CC and CXC subfamilies, whereas the XC and CX3C subfamilies are grouped in substantially smaller clusters. Mice and humans have ∼45 chemokines (3), which are involved, in sometimes extremely complex ways, in regulating *in vivo* leukocyte migration. Given the complexity of chemokine biology, it is common to simplify things by defining chemokines as being either homeostatic or inflammatory, according to the *in vivo* contexts in which they function (2, 4). Thus homeostatic chemokines regulate basal leukocyte trafficking to peripheral tissues and lymph nodes, whereas inflammatory chemokines are specifically involved in the attraction of inflammatory leukocytes to damaged or infected body sites. In the context of inflammatory responses, numerous chemokines are expressed simultaneously, and their overall amount and assortment orchestrate the migration of a variety of inflammatory leukocytes to the inflamed site. Effective resolution of inflammatory responses is dependent on appropriate and timely clearance of inflammatory chemokines from inflamed sites. In the absence of such clearance, the inflammatory response persists, and chronic pathologies evolve.

The chemokine scavenging receptor D6 (5, 6) is a prototypic member of the atypical chemokine receptor family. This family is defined on the basis of the inability of its members to mount classical receptor signaling responses following ligand binding (7–9). D6 is a promiscuous receptor with a binding selectivity for inflammatory CC chemokines (5, 6, 10, 11). D6 is an extremely efficient internalizer and degrader of inflammatory CC chemokines (12–15) and in this way contributes to the resolution of the inflammatory response. Mice deficient in D6 display a range of inabilities to resolve inflammatory responses in the tissues in which D6 is normally expressed. Thus D6-deficient mice display exaggerated cutaneous (16, 17), pulmonary (18), and gut (according to the specific model used (19, 20)) inflammatory responses, and in the context of the skin and gut, D6-deficient mice display enhanced tumorigenic programs in murine models of inflammation-dependent cancer development (20, 21). The major site of D6 expression is lymphatic endothelium (22), and we have hypothesized a role for lymphatic endothelial cell D6 in ensuring efficient drainage, and thus, removal of inflammatory chemokines and cytokines from inflamed sites (23, 24). In this way, we have suggested that the major role for D6 is to ensure the openness of the lymphatic drainage channels and that the exaggerated inflammatory response seen in D6-deficient mice relates to the inability of these mice to efficiently remove inflammatory cytokines and chemokines from inflamed sites. In keeping with its experimentally demonstrated role as a regulator of inflammatory responses, D6 has been shown to be broadly expressed in a range of inflammatory pathologies, suggesting a role in disease pathogenesis (25–28). Interestingly, D6 is expressed in a variety of cell types in inflammatory pathologies, including keratinocytes and peripheral blood leukocytes. It is therefore clear that D6 contributes to the resolution of the inflammatory response in a range of ways likely to involve both lymphatic endothelial cells as well as other cell types.

We have been particularly interested in examining the function of D6 in cutaneous inflammatory responses. Previously we have published that although WT mice display a mild and transient inflammatory response to phorbol ester (TPA)^3^ application, D6-deficient mice are unable to efficiently resolve this response (16) and develop a pathology that is similar, in numerous ways, to human psoriasis (26). The pathology develops in a characteristic temporal fashion, thus allowing the cellular and molecular basis to be defined. The purpose of the present study was to define the molecular signature of the cutaneous inflammatory pathology induced in D6-deficient mice with a view to understanding the precise roles for D6 in regulating inflammation. Here we report transcriptional evidence indicating that challenged D6-deficient mice mount a type I interferon-based response that is essential for the development of the cutaneous inflammatory pathology. These data further elucidate the mechanism of action of D6 and suggest a close association between D6 function and the suppression of type I interferon-dependent inflammatory responses.

## EXPERIMENTAL PROCEDURES

### 

#### 

##### Irritation of the Skin with the Phorbol Ester TPA

Dorsal skin of female 129-C57BL/6 mice (16) (8–12 weeks old) was shaved, and three applications of TPA (Sigma P1585, 50 μm, 150 μl/mouse) or acetone (150 μl/mouse) were applied to the skin at 24-h intervals. The cutaneous inflammation was left to develop for 1, 2, 4, and 6 days after the three TPA applications. Dorsal skin was removed from mice at each of these time points and stored in RNA*later*® (Invitrogen AM7020) for 24 h at 4 °C (for RNA purification) or fixed in formalin overnight at 4 °C for subsequent histological analysis.

##### RNA Extraction

Skin was removed from RNA*later*® and stored at −80 °C until processing. To extract RNA, back skin was ground into a powder in liquid N_2_, and RNA was extracted using TRIzol® and the PureLink® RNA kit (Ambion 12183018A) according to the manufacturer's instructions. RNA concentrations were quantified using the Nanodrop (Thermo Scientific) and stored at −80 °C.

##### Histology

Formalin-fixed skin samples were transferred to the tissue processor (Thermo Scientific) and progressively dehydrated over 20 h to xylene through successive concentrations of ethanol. Skins were embedded in paraffin wax, and 8-μm sections were cut, mounted onto Superfrost Slides (Fisher 12-550-15), and stored at 4 °C until required.

##### Hematoxylin and Eosin Staining

Paraffin-embedded skin sections were rehydrated with water and stained with hematoxylin and eosin according to standard procedures. Briefly, slides were stained with hematoxylin (2 min), dipped in 1% acid/alcohol twice, rinsed in water, immersed in Scotts Tap water substitute (30 s), rinsed in water, and stained with eosin (2 min). Slides were dehydrated to xylene, mounted in dibutyl phthalate xylene, and visualized on a light microscope (Carl Zeiss).

##### T Cell Staining

Paraffin-embedded skin sections were rehydrated with water, blocked with 20% horse serum in TBS-0.01% Tween 20 (TBST) for 30 min at room temperature, and incubated with a rabbit anti-human CD3 antibody (Dako) for 1 h at room temperature in TBST. Excess antibody was removed by washing twice in TBST, and staining was detected using the Dako Envision kit according to the manufacturer's instructions. The slides were dehydrated to xylene, mounted in DPX, and visualized on a light microscope (Carl Zeiss).

##### Microarray Analysis

Microarrays were performed using Affymetrix Mouse Genome 430 2.0 Exon Expression Arrays and subsequently analyzed using GeneSpring GX (Agilent). Three WT and three D6-deficient mice were used per time point over the four time points, and three acetone-treated controls were used for both D6-deficient and wild type mice. Microarray data were normalized using robust multiarray analysis and base-line-transformed to the median of the control samples (acetone-treated D6-deficient or WT mice) to allow visualization of both lowly and highly expressed genes. TPA-treated WT or D6-deficient mice were compared with their respective controls robust multiarray analysis normalization involved three steps: background correction (to remove noise), quantile normalization (to adjust for “chip to chip” variation), and summarization (to transform the data onto a log_2_ scale and remove outliers). To base-line transform the data, the median of the control samples, the log_2_ normalized intensity value for each gene in the TPA-treated samples was subtracted from the median normalized intensity value of the equivalent gene from the respective acetone-treated control sample (D6-deficient or WT mice). To remove noise and lowly expressed genes, the lower 20% of genes expressed were removed. Principal component analysis was performed to determine whether particular chips were outliers. Lists of significantly expressed genes were analyzed using gene ontology analysis to subsequently identify significantly changed families of genes. Hierarchical clustering within entities was also performed to identify gene expression patterns within the data. Ingenuity pathway analysis was used to identify potential differentially expressed pathways altered in D6-deficient mice compared with WT mice at each time point within gene lists identified using hierachical clustering. The data discussed in this publication are MIAME-compliant and have been entered into the NCBI Gene Expression Omnibus (accessible online under accession number GSE46889).

##### Real Time PCR

RNA (1 μg) was reverse transcribed to cDNA using the Quantitect reverse transcription kit (Qiagen 205311) according to the manufacturer's instructions. Gene expression was measured by absolute quantification compared with β-actin. DNA standards were made by cloning into TOPO TA cloning vector (Invitrogen 450641) using the primer sequences in Table 1. cDNA was diluted 1:5 and mixed with PerfeCTa SYBR green FastMix® (Quanta Biosciences 95072-250) and quantitative PCR primers (Table 1). The plate was run on an ABI 7900HT fast real time PCR System (ABI) under the conditions recommended for SYBR green by the manufacturer (Quanta Biosciences).

**TABLE 1:** **PCR primer sequences** Primer sequences were designed for measurement of gene copy number by quantitative PCR. Standards with a known copy number were generated by cloning into TOPO using the indicated primer sequences. The quantitative PCR was performed using the quantitative PCR primers and SYBR green. The copy number of each gene was measured using a standard curve generated using the cloned standards. Gene transcription was normalized to β-actin.

Gene	Forward (5′-3′)	Reverse (5′-3′)
**Quantitative PCR**		
*Irf7*	TGTTTGGAGACTGGCTATTGG	ATCCCTACGACCGAAATGCT
*Ifit2*	GAAAAAGAAAGCCCTCACCAA	GTTCCCCAAACTCCTGACAA
CXCL9	TTTCTCCCTCCCTCCCTTC	TTTTCACCCTGTTGGCTCT
β-Actin	CGTTGACATCCGTAAAGACC	CTGGAAGGTGGACAGTGAG

**Standards**		
*Irf7*	TGTGACCCTCAACACCCTA	GAGCCCAGCATTTTCTCTTG
*Ifit2*	CAGGAGAATGGAGGAGGTC	CTGAAACAAGCCCAAGACAAG
CXCL9	CCTCCTTGCTTGCTTACCAC	AACTCTGGCTCCCTTCC
β-Actin	GCTCTTTTCCAGCCTTCCTT	GCTCAGTAACAGTCCGCCTA

##### Neutralizing IFN-α and IFN-β in TPA-treated Mice

Neutralizing antibodies against IL-6 (rat anti-mouse), IL-20 (rat anti-mouse), IFN-α (rabbit anti-mouse), and IFN-β (rabbit anti-mouse), as well as isoptype and sera controls, were purchased from R&D Systems. Antibodies were injected intravenously into WT and D6-deficient mice (8–12 weeks old), 3 h before the first application of TPA (Sigma P1585, 50 μm, 150 μl/mouse). A further application of TPA was applied as normal 24 h later. The following day, the same amount of antibody or isotype control was injected intravenously, 3 h before the final application of TPA. Mice therefore received two doses of antibody or isotype control and three applications of TPA. Pathology was left to develop for 4 days, after which dorsal skin was taken for histology and quantitative PCR.

##### Statistical Analysis

The data were analyzed using unpaired *t* tests comparing D6-deficient with WT mice. *p* < 0.05 denotes significance.

## RESULTS

### 

#### 

##### D6-deficient Mice Display a Temporally Reproducible Pattern of Development of Exaggerated Cutaneous Inflammation

**FIGURE 1.**
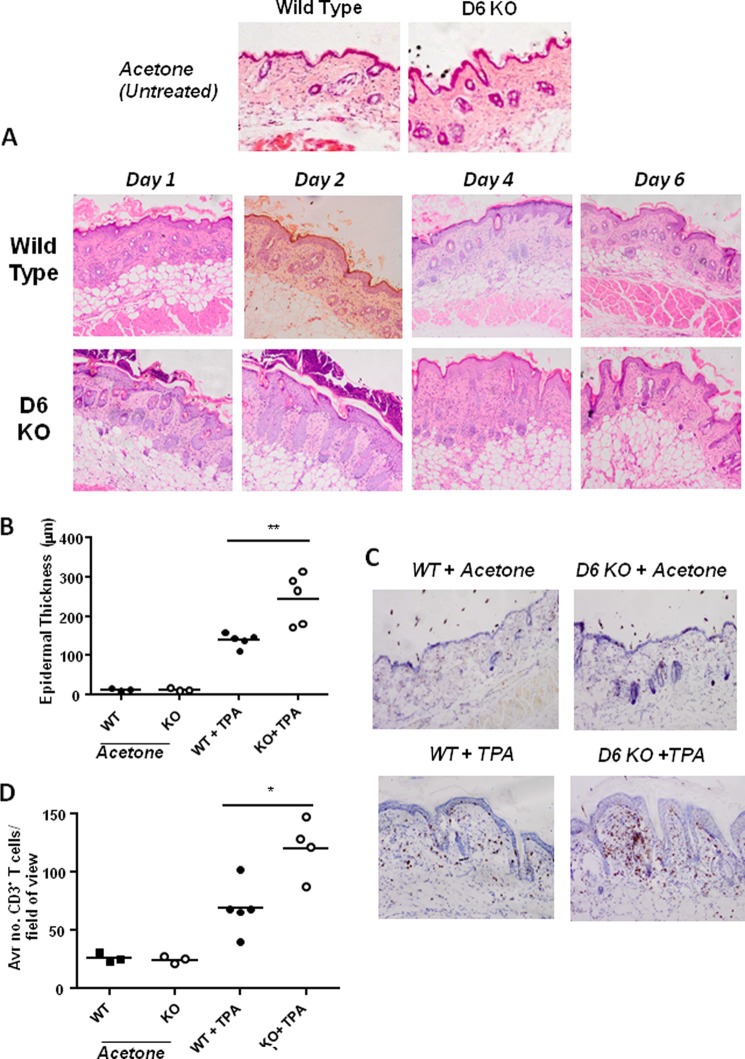
**D6 KO mice show an exaggerated cutaneous inflammatory response.** The shaved dorsal skins of D6-deficient or WT mice were treated with three applications of TPA (150 μl, 50 μm) or acetone (untreated mice), and the inflammatory pathology was left to develop for 1, 2, 4, and 6 days. *A*, histological analysis (H&E staining) of the development of the exaggerated cutaneous inflammatory pathology in D6-deficient (D6 KO) compared with wild type mice at the indicated time points after TPA treatment. Uninflamed skin (day 0) of acetone-treated wild type and D6 KO mice is also shown for comparison. *B*, assessment of the extent of cutaneous inflammation by quantification of epidermal thickness at the peak of the inflammatory pathology (day 4 after TPA treatment). Each point represents the mean of nine separate measurements. **, *p* < 0.001. *C*, demonstration of the exaggerated T cell accumulation in inflamed D6 KO mouse skins as revealed by CD3 staining of day 4 skins. *D*, quantitation of the T cell accumulation in resting (WT and D6 KO) and inflamed (day 4 WT+TPA and KO +TPA) WT and D6 KO skins. Each point represents the mean of nine separate measurements. *, *p* < 0.05.

We have previously published that D6-deficient mice display a markedly exaggerated response to mild cutaneous inflammatory stimuli (16). Because this represents a uniquely tractable model of impaired resolution of chemokine-driven inflammatory responses, we initiated a study aimed at investigating the transcriptomic basis for the cutaneous inflammatory pathology in D6-deficient mice with the hope that this may shed novel light on the molecular mechanisms of impaired resolution of cutaneous inflammation. Fundamental to this study was the observation that D6-deficient mice develop the inflammatory skin pathology in a defined temporal manner. As shown in Fig. 1*A*, uninflamed WT and D6-deficient mouse skin sections (day 0) are histologically indistinguishable. However, although WT mice develop a very mild and transient inflammatory response peaking at day 2 after TPA treatment, D6-deficient mice develop a much more profound inflammatory response, which is evident as early as day 1 after TPA treatment and which has not completely resolved even by day 6. Epidermal thickness, used as a quantitative surrogate measure of the extent of inflammation (Fig. 1*B*), confirmed the enhanced inflammatory response in D6-deficient mice at day 4 and also revealed that this is significantly greater than that seen with WT mice at the same time point. We have previously reported that a characteristic of the cutaneous inflammatory response developing in D6-deficient mice is the presence of T cells within the inflamed epidermis. As shown in Fig. 1*C*, and as enumerated in Fig. 1*D*, whereas WT mice show only a low level of T cell accumulation in the epidermis at day 4, D6-deficient mice show a highly significantly increased presence of such cells. This identical pattern of development of inflammation was seen in all mice used in this study, thus confirming the temporal reproducibility of the response.

##### Inflamed Skin of D6^−/−^ Mice Exhibit a Distinct Gene Expression Pattern

To investigate the transcriptional program underpinning the gross inflammatory response seen in D6-deficient mice, we harvested skin from TPA-treated D6-deficient and WT mice at the indicated time points, isolated RNA, and determined the differentially expressed genes using a microarray approach. Bioinformatic analysis of the data generated demonstrated that there were major differences in gene expression patterns between inflamed skin from D6-deficient and WT mice and that this was temporally regulated (Table 2). At base line, 48 genes were differentially regulated between D6-deficient and WT mice (13 up-regulated and 35 down-regulated; detailed in supplemental Table S1), although pathway analysis indicated that these genes represented no common biological process. These basal differences were taken into account in subsequent analyses by normalizing transcriptomic data from later time points for D6-deficient or WT TPA-treated samples to their respective untreated controls. In D6-deficient mice, over time, a total of 90 entities (30 up-regulated and 60 down-regulated) were altered at day 1 (supplemental Table S2), 406 (195 up-regulated and 211 down-regulated) were altered at day 2 (supplemental Table S3), 150 (49 up-regulated and 101 down-regulated) were altered at day 4 (supplemental Table S4), and 41 (20 up-regulated and 21 down-regulated) were altered at day 6 (supplemental Table S5). Thus the major differences in gene expression between D6-deficient and WT mice occurred at day 2, preceding the major differences in pathology, which were apparent at day 4 (Fig. 1*A*).

**TABLE 2:** **Number of differentially expressed genes at each time point** Number of differentially up- or down-regulated genes in inflamed D6-deficient skin compared to inflamed wild type skin at each time point. Genes, referred to as “entities,” differentially up- or down-regulated in D6-deficient skin compared to wild type skin at 0, 1, 2, 4, or 6 days after TPA application are enumerated. At each time point, entities significantly (*p* < 0.05) up- or down-regulated (fold change, >3) were selected. The total number of entities identified to be significantly changed at each time point is indicated.

Time	Total entities	Up-regulated	Down-regulated
0 days	48	13	35
1 days	90	30	60
2 days	406	195	211
4 days	150	49	101
6 days	41	20	21

##### Gene Ontology Analysis Reveals Differential Expression of Members of Specific Gene Families

**FIGURE 2.**
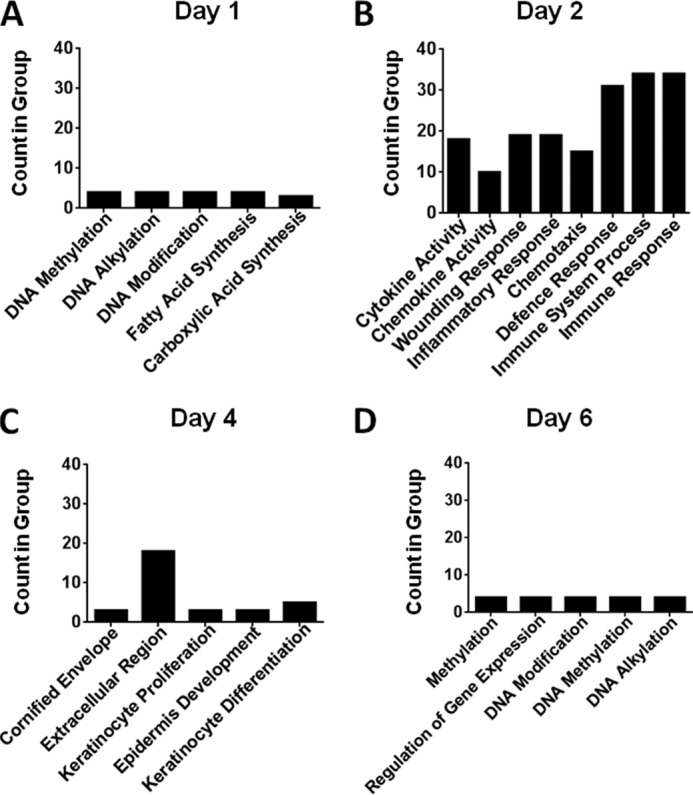
**Gene ontology analysis of the major families of genes displaying differential expression at the indicated time points.** Gene families displaying significantly altered expression (incorporating both up- and down-regulated genes) in D6 KO skin compared with wild type skins (>3-fold, *p* < 0.05). Gene expression differences at each time point: day 1 (*A*), day 2 (*B*), day 4 (*C*), and day 6 (*D*) were grouped into gene families using gene ontology analysis (Genespring). The number of genes within the list of significantly up- or down-regulated genes at each time point that fell into a particular gene family is indicated (*Count in Group*). Note the changes in the major altered gene families over the time course, particularly at day 2.

We next used gene ontology analysis to associate differentially expressed gene profiles with individual functional families by registering those families of genes that were significantly altered in D6-deficient, compared with WT, mice at each time point. Note that this analysis identifies gene families displaying significant alterations but does not rely on directionality and thus incorporates both up- and down-regulated genes in the analysis. We found that the number of genes that significantly fell into a particular family at day 1 was small, reflective of the relatively few genes (90 genes) differentially expressed at this time point. The majority of the genes differentially expressed at day 1 fell into families involving “DNA methylation” and “alkylation,” characteristic of skin turnover (Fig. 2*A*). However, the large number of genes differentially expressed at day 2 (406 genes) were preferentially associated with alternative gene families implicated in inflammatory responses such as “immune response,” “defense response,” “immune system process,” “inflammatory response,” and “response to wounding” (Fig. 2*B*). These differences were reflected in significant alterations in the temporal pattern and intensity of chemokine and chemokine receptor expression in the D6-deficient mice at this time point (supplemental Fig. S1, *A* and *B*). Specifically, and in contrast to WT mice, numerous inflammatory chemokines were overrepresented at day 2 in the D6-deficient mice. There was also enhanced representation of the inflammatory CC chemokine receptors CCR1, CCR2, and CCR5 (but not CCR3), indicative of increased accumulation of inflammatory cells bearing these receptors (supplemental Fig. S2). Notably, there was a significant reduction in expression of CCL20 as well as the CCR4 ligands CCL17 and CCL22 in D6-deficient mice compared with WT mice at this time point, indicating a potential shift away from atopic responses toward a more straightforward inflammatory response (supplemental Fig. S1*B*). In contrast to the major representation of inflammatory gene families at day 2, we found, after 4 days, that the major families of genes altered were those implicated in “keratinocyte differentiation,” “proliferation,” and “epidermal development” (Fig. 2*C*), matching with the histology (Fig. 1*A*), which indicated that the major differences in epidermal thickness were apparent at this time point (Fig. 1, *A* and *B*). These transcriptional alterations are reflected in marked differences in the expression of a broad range of genes involved in epidermal cell proliferation and cutaneous remodelling. Specifically, as shown in supplemental Fig. S3, there were differences in expression of a range of keratin genes indicative of the aberrant epidermal differentiation apparent in the inflamed D6-deficient skins. Furthermore, there was down-regulation of a large number of members of the *Lce1* class of late cornified envelope genes, which encode proteins that have been strongly implicated as being involved in the development of a range of cutaneous inflammatory pathologies (29, 30), most notably psoriasis. Also evident in supplemental Fig. S3 is the down-regulation of the epidermal genes Involucrin (*Ivl*) and Fillagrin (*Flg*). Together, these gene differences reflect the marked alterations in epidermal proliferation and differentiation in the D6-deficient mice. At day 6, the differences in gene expression between D6-deficient and wild type mice had largely been removed and again were restricted to genes involved in basic cellular processes (Fig. 2*D*).

##### Inflamed D6-deficient Mouse Skin Is Characterized by Altered Expression of a Range of Key Inflammatory Cytokines

**FIGURE 3.**
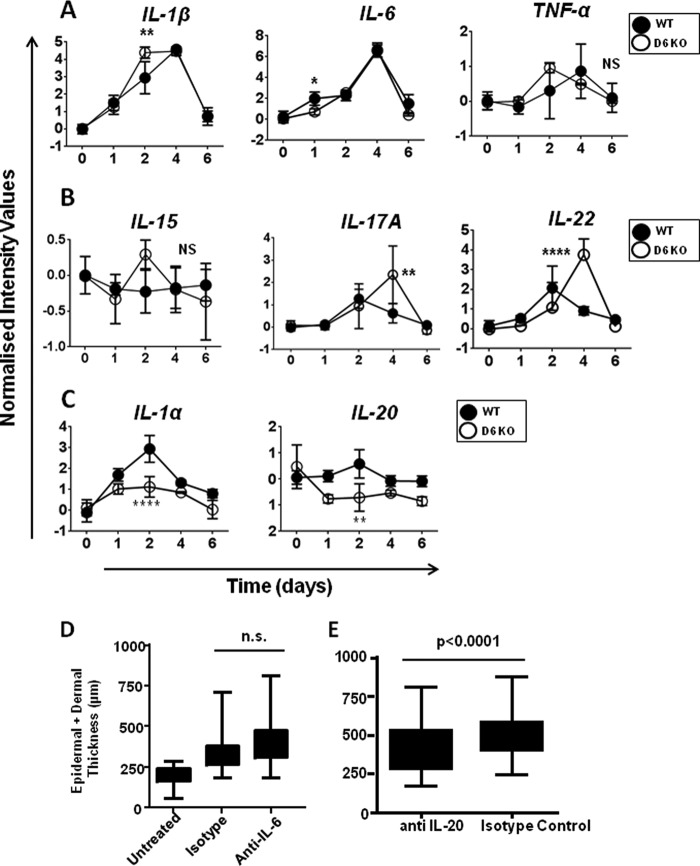
**Evidence of differential cytokine transcript levels in D6-deficient mice.** Kinetics of cytokine expression, over time, within the back skin of TPA treated wild type (*filled circles*) and D6 KO mice (*open circles*) are indicated in the profile plots (*A–C*). The data are expressed as normalized intensity values (log_2_; *y* axis) over time (days; *x* axis). *A*, profile plots indicating expression levels of IL-1β, IL-6, and TNF-α over the time course of the study in both WT and D6 KO skins. None of these cytokines displayed significant differences in the magnitude of induced expression in WT and KO mice, but differences in temporal expression were noted. *, *p* < 0.05; **, *p* < 0.01. *B*, profile plots indicating expression levels of IL-15, IL-17A, and IL-22 over the time course of the study in both WT and KO skins. These cytokines displayed enhanced differences in gene expression in KO mice compared with WT mice. **, *p* < 0.01; ****, *p* < 0.0001. *C*, profile plots indicating expression levels of IL-1α and IL-20 over the time course of the study in both WT and KO skins. These cytokines displayed reduced differences in gene expression in KO mice compared with WT mice. **, *p* < 0.01; ****, *p* < 0.0001. *D*, KO mouse skin was either left untreated or subjected to TPA-induced inflammation in the presence or absence of a systemically administered IL-6 neutralizing antibody. Skin thickness (epidermal plus dermal) was measured as an indication of the extent of cutaneous inflammation. The results demonstrate no significant effect of blocking interleukin-6 on development of the cutaneous inflammatory pathology. *n.s.*, not significant. *E*, skin thickness (epidermal plus dermal) measurements of KO mice subjected to TPA inflammation demonstrating a significant effect of systemic anti-IL-20 administration on the development of the cutaneous inflammatory pathology.

We next examined the differential expression of a range of cytokines involved in inflammatory responses and of known relevance to cutaneous inflammatory disorders (31–33). As shown by the profile plots in Fig. 3, a number of patterns was observed. First, some inflammatory cytokines displayed identical levels of transcriptional induction in inflamed WT and D6-deficient mouse skins (Fig. 3*A*) including IL-1β, IL-6, and TNF. However, whereas the temporal expression patterns of IL-6 were the same in WT and D6-deficient skins, IL-1β was induced earlier in the inflammatory process in D6-deficient skin compared with WT skins (*p* < 0.01), and TNF displayed a similar, albeit not significant, trend. IL-17A (*p* < 0.01) and IL-22 (*p* < 0.0001) were overexpressed in the D6-deficient mouse skins compared with WT skins, as was IL-15, but this difference did not reach statistical significance (Fig. 3*B*). Finally, other cytokines displayed markedly reduced expression in D6-deficient skins (Fig. 3*C*), including IL-1α (p < 0.0001) and IL-20 (*p* < 0.01). Interestingly, overexpression of IL-17A and IL-22 peaked at day 4, which contrasts with the peak expression of these two cytokines in WT mice at day 2, suggesting that their expression is maintained inappropriately in D6-deficient mice. We have previously reported that the pathology that develops in the D6-deficient mice can be blocked using antibodies, or other blocking agents, for TNF, IL-1β, IL-15, and IL-17A (16, 34), and this is in keeping with the differential expression of these cytokines demonstrated in Fig. 3. Interestingly, whereas IL-6 may also be regarded as a key regulator of inflammatory responses, it is does not display differential peak expression in wild type and D6-deficient mice, and accordingly neutralization of IL-6 had no impact on the development of the cutaneous inflammatory pathology in D6-deficient mice (Fig. 3*D*). In contrast, IL-20, which is overexpressed in inflamed WT but not D6-deficient mice, appears to be, at least partially, a contributor to the inflammatory response because neutralization significantly reduced the extent of the inflammatory response observed (Fig. 3*E*). Overall these data suggest differential expression of some cytokines but that differential expression patterns do not necessarily relate to the importance of cytokines for driving the inflammatory pathology in D6-deficient mice.

##### Type I IFN-related Genes Represent One of the Most Significantly Up-regulated Families of Genes

Notably, in addition to the variable differential expression of a variety of inflammatory cytokines, one consistency apparent from gene ranking studies was the overexpression of genes belonging to, or regulated by, the type I IFN pathway at day 2 in the D6-deficient mice (Table 3). The differentially expressed type 1 IFN pathway genes included *Ifit2*, *Irf7*, and other type I IFN-induced genes such as *Ifit44*, *Rsad2*, *Ifit2*, *Irf7*, and *Mx1*, which were up-regulated up to 16-fold in D6-deficient mice, compared with WT mice (Table 3, *p* < 0.0001).

**TABLE 3:** **Differentially expressed type I IFN pathway genes in D6−/− skins at day 2** Top up-regulated genes at day 2 after TPA application in the back skin of D6-deficient mice compared to wild type mice. The most highly up-regulated genes in D6-deficient skin compared to wild type skin at day 2 after TPA application are shown. Genes were identified using “volcano plots,” where genes significantly (*p* < 0.05) up-regulated (fold change, >3) were selected.

Probe set identifier	Gene symbol	Fold change	*P* value
1450783_at	*Ifit1*	15.67	0.00
1421009_at	*Rsad2*	12.88	0.00
1423555_a_at	*Ifit44*	12.53	0.03
1418293_at	*Ifit2*	12.35	0.00
1424339_at	*Oasl1*	12.25	0.00
1417244_a_at	*Irf7*	11.9	0.01
1421008_at	*Rsad2*	11.1	0.00
1427381_at	*Irg1*	10.73	0.04
1453196_a_at	*Oasl2*	9.73	0.00
1436058_at	*Rsad2*	9.45	0.00
1424775_at	*Oas1a*	9.3	0.01
1449025_at	*Ifit3*	8.84	0.00
1418191_at	*Usp18*	7.74	0.00
1418930_at	*Cxcl10*	6.37	0.00
1439114_at	*Ddx60*	6.08	0.00
1440865_at	*Ifitm6*	5.67	0.00
1451777_at	*Ddx60*	5.6	0.00
1451426_at	*Dhx58*	5.39	0.00
1425065_at	*Oas2*	4.95	0.01
1440866_at	*Eif2ak2*	4.05	0.00
1425374_at	*Oas3*	3.97	0.02
1419569_a_at	*Isg20*	3.96	0.00
1417292_at	*Ifi47*	3.82	0.01
1452348_s_at	*Ifi204*	3.61	0.04
1422006_at	*Eif2ak2*	3.6	0.00
1419603_at	*Ifi204*	3.48	0.04
1426278_at	*Ifi27l2a*	3.46	0.00
1436562_at	*Ddx58*	3.37	0.00
1421911_at	*Stat2*	3.37	0.00
1419043_a_at	*Iigp1*	3.22	0.04
1418126_at	*Ccl5*	3.19	0.02
1424254_at	*Ifitm1*	3.16	0.05
1450403_at	*Stat2*	3.16	0.00
1425405_a_at	*Adar*	3.04	0.00

##### Hierachical Clustering and Ingenuity Pathway Analyses Confirm That the Type I IFN Pathway Is Significantly Up-regulated in D6-deficient Mice

**FIGURE 4.**
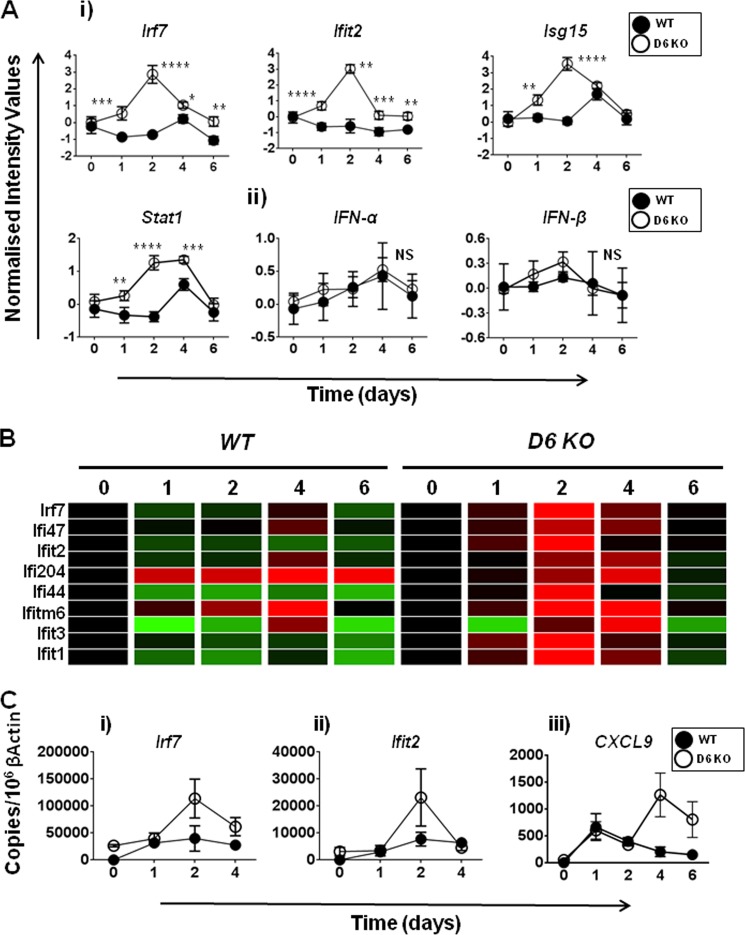
**The type I interferon pathway is overrepresented in D6 KO mice.** *A*, *panel i*, profile plots demonstrating differences in the levels of induction of type I interferon pathway genes *Irf7*, *Ifit2*, *Isg15*, and *Stat1* in WT (*filled circles*) and KO (*open circles*) inflamed mouse skins. *Panel ii*, profile plots revealing the similarity in the induced expression levels of IFN-α and IFN-β in WT and KO skins over the course of the induction of inflammation. In both *panels i* and *ii*, the data are expressed as normalized intensity values (log_2_; *y* axis) over time (days; *x* axis). *, *p* < 0.05; **, *p* < 0.01; ***, *p* < 0.001; ****, *p* < 0.0001. *B*, heat map analyses of the differential expression of a select group of type I interferon pathway genes over the course of the study in WT and D6-deficient (KO) mice after TPA treatment. *Black*, no change; *green*, down-regulated; *red*, up-regulated. The time points are indicated along the *top* of the heat map (for *WT*, *0* indicates WT day 0, 1 indicates WT day 1, etc.). *C*, confirmatory PCR demonstrating increased expression of type I interferon pathway genes in inflamed D6 KO compared with WT skins. *Panel i*, *Lrf7. Panel ii*, *Ifit2. Panel iii*, CXCL9. These PCR analyses were performed on skin samples isolated from an experiment separate from that used to generate the array data. The data are shown as absolute copy number of each gene compared with 10^6^ copies of β-actin.

To provide further support to the hypothesis that the type I IFN pathway was significantly up-regulated in D6-deficient mice at day 2, we performed hierachical clustering of the genes differentially regulated at day 2, to identify clusters of genes that were coexpressed in these mice (supplemental Fig. S4). The differentially expressed genes were plotted over the time frame of the study for both D6-deficient and WT mice to identify their patterns of expression. We found that the cluster containing the 34 genes listed in Table 3 was significantly elevated at day 2 in D6-deficient mice and was also sustained at day 4 (supplemental Fig. S4*A*). Analyzing the full list of type I IFN pathway genes using ingenuity pathway analysis demonstrated the interactive nature of the differentially expressed components of the cluster (supplemental Fig. S4*B*). In contrast, this family of genes was only up-regulated at day 4 in WT mice and in a less comprehensive manner. This suggests, overall, that this family of genes was expressed earlier and more fully in D6-deficient, compared with WT, mice. Interestingly, these differences in expression of IFN pathway genes such as *Irf7*, *Ifit2*, *Isg15*, and *Stat1* were apparent (Fig. 4*A*, *panel i*), despite there being no significant alterations in the temporal expression patterns of either IFNα or IFNβ (Fig. 4*A*, *panel ii*). We also analyzed IFNα and IFNβ protein levels in inflamed D6-deficient mouse skin, but they were below the levels of detection. The possible mechanisms whereby lack of alterations in IFNα and IFNβ transcript levels leads to the exaggerated type I IFN family gene expression in D6-deficient mice are addressed, in more detail, under “Discussion.” A number of the other overexpressed type I IFN pathway genes showing the most specific elevation in D6-deficient, compared with WT, mice are shown in the heat map in Fig. 4*B*. To confirm that the IFN pathway was up-regulated in the skin of D6-deficient, compared with WT, mice, quantitative PCR was performed for *Irf7*, *Ifit2*, and *CXCL9* using RNA derived from a separate skin inflammation study (Fig. 4*C*). This analysis confirmed the up-regulation of *Irf7*, *Ifit2*, and CXCL9 in the skin of D6-deficient mice 2 days after termination of TPA treatment. There were some differences noted in the magnitude of induction of these three genes between the microarray and PCR analyses. However, importantly, the expression “trends” were maintained and confirmed in these two separate experiments. Thus, overall, these data demonstrate the presence of an early and pronounced type I IFN gene expression signature in the inflamed skins of D6-deficient mice.

##### The Type I IFN Pathway Is Involved in the Development of the Cutaneous Inflammatory Pathology in D6-deficient Skin

**FIGURE 5.**
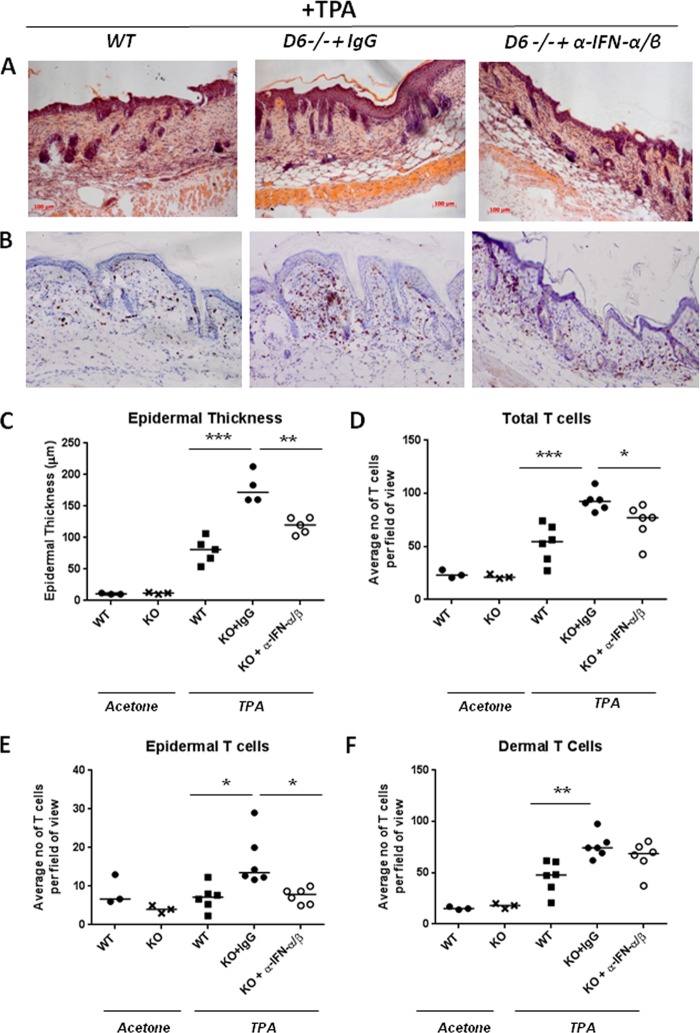
**The pathology that develops in D6-deficient mice is dependent on the type I interferon pathway.** D6-deficient (D6^−/−^) or WT mice were injected intravenously with rabbit anti-mouse IFN-α and rabbit anti-mouse IFN-β or a rabbit IgG control 3 h prior to the first application of TPA (150 μl, 50 μm) to the shaved dorsal skin. The same dose of TPA was applied 24 h later. The next day, 3 h before the last TPA application, the mice were injected with a final dose of rabbit anti-mouse IFN-α and rabbit anti-mouse IFN-β or rabbit IgG control. The inflammatory pathology was left to develop for 4 days following the final TPA application, after which skins were processed for histological analysis. *A*, H&E staining of skins at day 4 after TPA application indicating development of inflammatory pathology in D6^−/−^ mice and the amelioration of the pathology by antibodies to type I interferons. *B*, CD3 T cell staining demonstrating increased T cell recruitment into the D6^−/−^ mouse skins and its suppression by antibodies to type I interferons. *C*, quantification of epidermal thickness at day 4 demonstrating a significant reduction in the presence of neutralizing antibodies to type I interferons. *D*, quantification of the number of CD3-positive T cells in the skins at day 4 demonstrating a significant reduction in D6^−/−^ (KO) mice treated with neutralizing antibodies to type I interferons. *E*, specific quantification of T cells within the epidermal compartment. *F*, specific quantification T cells within the dermal compartment. In *C–F*, each point is representative of a mean of nine separate measurements per mouse.

We hypothesized, on the basis of the microarray data, that the inflammation observed in the skin of D6-deficient mice was, at least in part, dependent on the activities of type 1 IFNs within the skin (note that IFNγ plays no apparent role in the pathology; data not shown). To formally test this, neutralizing antibodies to IFNα and IFNβ were injected intravenously prior to and during TPA treatment of WT and D6-deficient mice. Importantly, although antibody blockade of type I IFN activity had a modest effect on inflammation in WT mice, as measured by total skin thickness (supplemental Fig. 5*A*), this did not reach statistical significance and was not reflected in the other measure of cutaneous inflammation, epidermal thickness (supplemental Fig. S5*B*). In contrast, we found that, after 4 days, anti-IFN antibody treatment was associated with a significant reduction in the inflammatory cutaneous pathology in D6-deficient mice as demonstrated by decreased epidermal thickness (Fig. 5, *A* and *C*). In addition, a modest but significant reduction in total cutaneous T cells was observed in the anti-IFN antibody-treated mice (Fig. 5, *B* and *D*). Importantly, and in keeping with the preferential accumulation of T cells in the epidermal compartment in inflamed D6-deficient mouse skin (16), the difference in T cells was largely accounted for by a reduced accumulation in the epidermal compartment (Fig. 5*E*). No difference in dermal T cell accumulation was noted (Fig. 5*F*). For both total T cells and epidermal T cells, anti-IFN antibody treatment reduced the levels to those seen in inflamed wild type skin. Thus the differential expression of type I interferon response genes reflects the importance of this pathway for the development of the cutaneous inflammatory response in D6-deficient mice.

## DISCUSSION

In the context of cutaneous inflammatory responses, D6-deficient mice develop an exaggerated inflammatory pathology that bears many similarities to human psoriasis (16). Furthermore, D6 is differentially expressed in psoriasis in a manner indicative of a role in pathogenesis (34). The aim of the present study was to define the molecular anatomy of this response and to gain insights into the molecular basis for the impaired resolution of inflammation apparent in these mice. The data presented demonstrate clear transcriptional differences in inflamed skins of WT and D6-deficient mice. These differences are, in general, indicative of accelerated and exaggerated inflammatory responses in the D6-deficient mice. At later time points, the transcriptional signature is indicative of alterations to epidermal differentiation and remodelling, which is very much in keeping with the histology reported in this and previous (16, 34) papers indicating massive hyperproliferation of the epidermis and aberrant differentiation in the D6-deficient mice. The transcriptomic patterns therefore closely reflect the pathology.

In terms of cytokine regulation of the development of the inflammatory response in D6-deficient mice, a number of expression patterns are observed. First, some cytokine transcripts, such as IL-6, display no differences between WT and D6-deficient mice and antibodies to IL-6 fail to ameliorate the inflammatory pathology, indicating no active involvement of the cytokine in the pathology. However, other cytokines demonstrate either prolonged expression, as in the case of IL-1β, or enhanced expression in the case of IL-15, IL17A, and IL-22. Our previous results indicate that IL-1β, IL-15, and IL-17A, along with TNF, are essential players in the pathology that develops (16, 34). One important question that emerges from these observations is why so many different cytokines can be pivotal to the development of this inflammatory pathology. These observations are not unusual, and numerous diverse cytokines have been demonstrated to play important roles in individual murine inflammatory disease models. Perhaps the most comprehensive exemplar of this is collagen-induced arthritis, in which a very broad range of cytokines has been shown to be essential for development of the pathology (35, 36). Our interpretation of this is that it suggests that pathological development is dependent on a network of cytokines and not on individual cytokines and that interfering with any arm of this network is sufficient to block development of inflammatory pathology. This therefore has implications for therapy and suggests that there may be numerous distinct intervention points in each inflammatory pathology.

One of the most striking features of our microarray data is that it strongly highlights rapid onset and elevated expression of transcriptional differences in genes belonging to the type I interferon signaling pathway. In the context of D6-deficient mice as a model of psoriasis, this is of importance because type I interferons (produced by plasmacytoid dendritic cells) have been clearly demonstrated to be involved in the human pathology (37–40). Importantly, a recent microarray analysis of psoriatic skin in comparison to nonlesional skin has again highlighted the type I interferon pathway is being at play, and therefore of therapeutic value, in human psoriasis (41). Indeed, of the top 50 most up-regulated type I interferon-inducible genes identified in psoriatic lesions, 25 are also up-regulated in the D6-deficient mice, further supporting the notion that a similar type I interferon pathway is active in this model. The ability of antibodies to type I interferons to suppress the development of the pathology in D6-deficient mice is therefore not entirely surprising. However, it is interesting that this is seen despite the fact that neither IFNα or IFNβ display increased transcript levels in D6-deficient mice. The question arises therefore how cytokines with no enhanced transcriptional profiles can be differentially driving pathology in wild type and D6-deficient skin. We propose that the answer to this relates to our model of D6 function (23, 24). This model hypothesizes that the failure of resolution of inflammation in D6-deficient mice relates to expression of this chemokine scavenging receptor on lymphatic endothelial cells. Specifically, in the absence of D6, inflammatory chemokines congregate around the lymphatic endothelial surfaces and trigger inappropriate association of numerous inflammatory leukocytes with the lymphatic surface. This congests the lymphatic system and impairs lymphatic drainage. A consequence of this is that inflammatory chemokines that drive inflammatory leukocyte recruitment, as well as the cytokines that induce inflammatory chemokines such as TNF and the type I IFNs, drain inefficiently from inflamed sites in D6-deficient mice. This results in prolonged inflammatory cytokine activity, and leukocyte accumulation, at such inflamed sites. Thus we propose that although IFNα and IFNβ are expressed at similar levels in wild type and D6-deficient mice, they are not removed as efficiently from D6-deficient skin and therefore continue to drive aspects of the pathology. In this way, we believe, they contribute to the development of the psoriasiform pathology. Interestingly, we have previously reported that D6 expression is increased in both keratinocytes and lymphatic endothelial cells following exposure to type I interferons (26, 34). This suggests, therefore, that the interferon pathway not only drives inflammation but also up-regulates D6 as feedback to limit this response. This further explains the exaggerated type I interferon-dependent inflammatory response in D6-deficient mice.

In summary, therefore, these transcriptomic data demonstrate strong transcriptional similarities between the D6-deficient mouse model of cutaneous inflammation and human psoriasis. Our data are therefore important in that they further implicate D6 in the pathogenesis of psoriasis and provide an essential link between reduction in D6 expression, as noted in psoriatic plaques (26), and the development of type I IFN-driven pro-psoriatic inflammatory responses. In addition, our data suggest that, because D6 is transcriptionally up-regulated by type I IFNs, this axis represents a negative feedback loop restricting the chemokine aspect of type I IFN driven inflammatory responses.

## Footnotes

3 The abbreviation used is:

TPA: 12-*O*-tetradecanoylphorbol-13-acetate.
